# Optimizing sgRNA structure to improve CRISPR-Cas9 knockout efficiency

**DOI:** 10.1186/s13059-015-0846-3

**Published:** 2015-12-15

**Authors:** Ying Dang, Gengxiang Jia, Jennie Choi, Hongming Ma, Edgar Anaya, Chunting Ye, Premlata Shankar, Haoquan Wu

**Affiliations:** Department of Biomedical Sciences, Paul L. Foster School of Medicine, Texas Tech University Health Sciences Center El Paso, El Paso, TX 79905 USA

## Abstract

**Background:**

Single-guide RNA (sgRNA) is one of the two key components of the clustered regularly interspaced short palindromic repeats (CRISPR)-Cas9 genome-editing system. The current commonly used sgRNA structure has a shortened duplex compared with the native bacterial CRISPR RNA (crRNA)–transactivating crRNA (tracrRNA) duplex and contains a continuous sequence of thymines, which is the pause signal for RNA polymerase III and thus could potentially reduce transcription efficiency.

**Results:**

Here, we systematically investigate the effect of these two elements on knockout efficiency and showed that modifying the sgRNA structure by extending the duplex length and mutating the fourth thymine of the continuous sequence of thymines to cytosine or guanine significantly, and sometimes dramatically, improves knockout efficiency in cells. In addition, the optimized sgRNA structure also significantly increases the efficiency of more challenging genome-editing procedures, such as gene deletion, which is important for inducing a loss of function in non-coding genes.

**Conclusions:**

By a systematic investigation of sgRNA structure we find that extending the duplex by approximately 5 bp combined with mutating the continuous sequence of thymines at position 4 to cytosine or guanine significantly increases gene knockout efficiency in CRISPR-Cas9-based genome editing experiments.

**Electronic supplementary material:**

The online version of this article (doi:10.1186/s13059-015-0846-3) contains supplementary material, which is available to authorized users.

## Background

The clustered regularly interspaced short palindromic repeats (CRISPR) system has recently been developed into a powerful genome-editing technology [1–6]. This system is composed of two components: the nuclease Cas9 and the guide RNA. After maturation, the native type-II CRISPR guide RNA is composed of a 42-nucleotide CRISPR RNA (crRNA) and an 89-nucleotide transactivating crRNA (tracrRNA) [6] (Figure S1a in Additional file 1). Jinek et al. [6] systematically studied the minimal sequence requirement of the guide RNA in vitro and linked two minimal sequences together to create the short-version single-guide RNA (sgRNA; +48 nucleotides; Figure S1b in Additional file 1). However, a longer version of the sgRNA (+85 nucleotides), which is 37 nucleotides longer at the 5’ end (Figure S1c in Additional file 1), was shown to be much more efficient [7–9] and is now commonly used. This commonly used sgRNA has a shortened duplex compared with the native guide RNA (Figure S1a, c in Additional file 1). In addition, there is a continuous sequence of Ts, which is the pause signal for RNA polymerase III; this signal could potentially reduce transcription efficiency and knockout efficiency. Hsu et al. [9] showed that changing these two elements did not have a significant effect on knockout efficiency and concluded that the sgRNA (+85 nucleotides) without mutations and duplex extension is the most active sgRNA architecture. However, Chen et al. [10] reported that sgRNAs with a mutated continuous sequence of Ts and extended duplex significantly enhance the imaging efficiency of a dCas9 (a mutated version of Cas9 lacking nickase activity)–green fluorescent protein (GFP) fusion protein in cells, suggesting that changing these two elements enhances dCas9 binding to target sites and might also increase the knockout efficiency of Cas9. In this study, we systematically investigated the effect of changing these two elements on knockout efficiency and found that, overall, extending the duplex and mutating the continuous sequence of Ts significantly improved knockout efficiency.

## Results

**Fig. 1:**
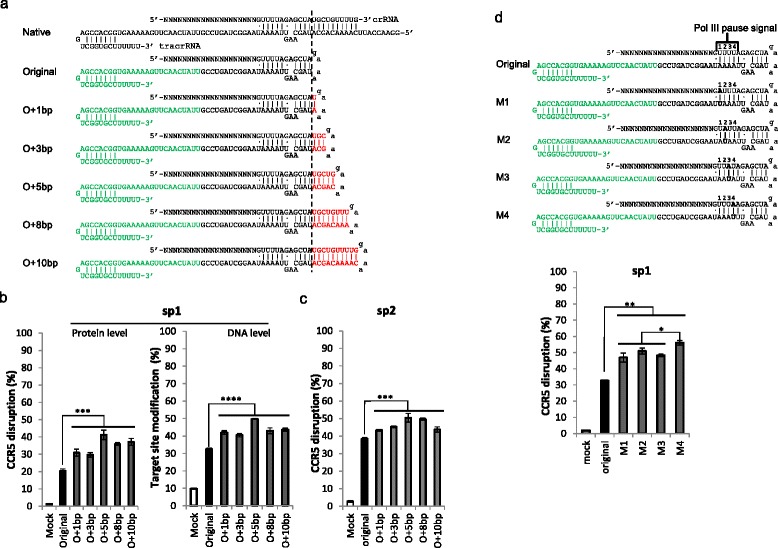
Knockout efficiency can be increased by extending the duplex and disrupting the continuous sequence of Ts. **a** The duplex extension. *Green* indicates the 3’ 34 nucleotides, which are not required for sgRNA functionality in vitro but are required in cells; *red* indicates the extended base pairs. **b** Extension of the duplex increased knockout efficiency. Constructs harboring sgRNAs targeting the *CCR5* gene were co-transfected with a Cas9-expressing plasmid into TZM-bl cells. An sgRNA targeting the HIV genome served as mock control. The GFP-positive cells were sorted out 48 hours after transfection, and the gene modification rates were determined at the protein and DNA levels, respectively. Protein level disruption: the expression of CCR5 was determined by flow cytometry analysis. The raw data are shown in Figure S2 in Additional file 1. DNA level modification rate: the genomic DNA was extracted, and the target sites were amplified and deep-sequenced with a MiSeq sequencer. The raw data are provided in Additional file 2. **c** The experiment in (**b**) at the protein level was repeated for another sgRNA, sp2. The difference with (**b**) is that the cells were not sorted, but the *CCR5* disruption rate was measured in GFP-positive cells. The raw data are shown in Figure S2 in Additional file 1. **d** Mutation of the RNA polymerase (*Pol III*) pause signal significantly increased knockout efficiency. The mutated nucleotides are shown in *bold*. The raw data are shown in Figure S3 in Additional file 1. The graphs represent biological repeats from one of three independent experiments with similar results, shown as mean ± standard deviation (*n* = 3). Significance was calculated using Student's *t*-test: *P < 0.05; **P < 0.01; ***P < 0.005; ****P < 0.001. *O* original, *M* mutant

The current most commonly used sgRNA design has the duplex shortened by 10 bp compared with the native crRNA–tracrRNA duplex (Fig. 1a), which does not seem to reduce its functionality in vitro [6]. Hsu et al. [9] also showed that extending the duplex appeared to have no effect on knockout efficiency in cells. However, Chen et al. [10] showed that extending the duplex significantly enhances imaging efficiency of the dCas9–GFP fusion protein in cells. We suspected that extending the duplex might increase knockout efficiency in cells. To test this hypothesis, we extended the duplex in two sgRNAs targeting the *CCR5* gene, as shown in Fig. 1a, and determined the knockout efficiency of these mutants in TZM-bl cells. Extending the duplex by 1, 3, 5, 8, or 10 bp significantly increased the knockout efficiency in both sgRNAs tested, and extending the duplex by 5 bp appeared to yield the highest efficiency at the protein level (Fig. 1b; Figure S2 in Additional file 1). The modification rate at the DNA level was also confirmed by deep sequencing of target sites (Additional file 2), and the results correlated well with the results determined at the protein level (Fig. 1b; Figure S2 in Additional file 1). Since measuring the modification rate by deep sequencing is more expensive and labor intensive, we mainly relied on fluorescence-activated cell sorting (FACS) to determine the *CCR5* disruption rate in this study. When the effect of extending the duplex was tested for another sgRNA (sp2), the results were consistent with those for sp1 (Fig. 1c; Figure S2 in Additional file 1). Thus, extending the duplex appears to increase the knockout efficiency of the CRISPR-Cas9 system.

Because the continuous sequence of Ts after the guide sequence is the pause signal for RNA polymerase III [11], the effect of its disruption in sgRNAs has been previously studied [9, 10]. We suspected that mutating the continuous sequence of Ts might also improve knockout efficiency in cells. Accordingly, we mutated this sequence at different positions and determined the knockout efficiency of the mutants (Fig. 1d; Figure S3 in Additional file 1). The knockout efficiency was increased in all mutants, and the mutation at position 4 had the greatest effect.

**Fig. 2:**
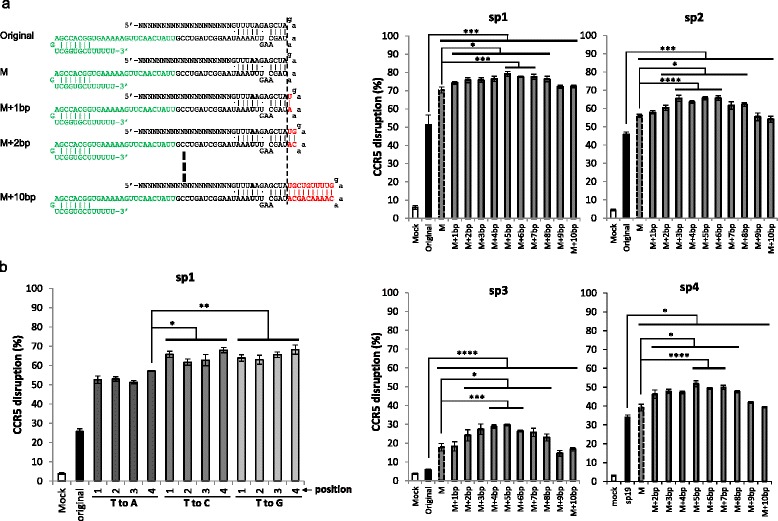
Knockout efficiency can be further increased by combining duplex extension with disruption of the continuous sequence of Ts. **a** The effect of duplex extension when mutating the fourth T to an A in four sgRNAs. The raw data are shown in Figure S4 in Additional file 1. **b** The effect of mutation of Ts at the indicated positions to A, C, or G when also extending the duplex by 5 bp. The raw data are shown in Figure S5 in Additional file 1. The graphs represent biological repeats from one of three independent experiments with similar results, shown as mean ± standard deviation (*n* = 3). Significance was calculated using Student's *t*-test: *P < 0.05; **P < 0.01; ***P < 0.005; ****P < 0.001. *M* mutant

Next, we systematically investigated the effect of extending the duplex while mutating the fourth T in the sequence of Ts (Fig. 2a; Figure S4 in Additional file 1). Consistent with the result shown in Fig. 1b, mutating the fourth T increased the knockout efficiency significantly for all four sgRNAs tested (Fig. 2a). On top of the increase due to mutation, extending the duplex also increased the knockout efficiency, reaching a peak at around 5 bp but then declining with longer extensions, although the pattern appears to be slightly different for different sgRNAs (Fig. 2a), which is consistent with Chen et al.’s results showing that modifying both elements significantly enhances the imaging efficiency of a dCas9–GFP fusion protein in cells [10].

We previously tested the effect of mutating T→A on knockout efficiency without extending the duplex (Fig. 1c). Next, we also wanted to test the effect of mutating T→A, C, or G while also extending the duplex. Consistent with previous observations, mutations at position 4 generally had the highest knockout efficiency, although mutating T→C at position 1 had a similar effectiveness. In addition, mutating T→C or G generally had higher knockout efficiency than mutating T→A at various positions (Fig. 2b; Figure S5 in Additional file 1). Thus, mutating T→C or G at position 4 yielded the highest knockout efficiency.

**Fig. 3:**
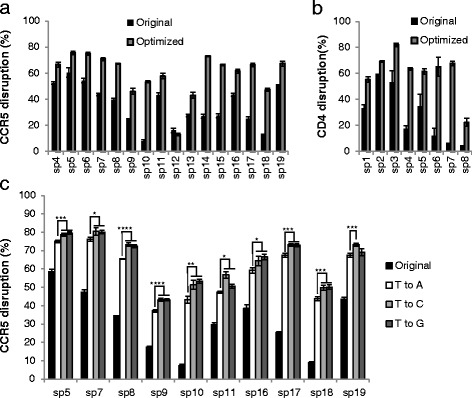
The optimized sgRNA structure is superior to the original version. **a** *CCR5* knockout efficiency was determined for the indicated sgRNAs targeting *CCR5* with either an optimized sgRNA structure or the original structure. The knockout efficiency was determined in the same way as in Fig. 1b. The raw data are sown in Figure S6 in Additional file 1. **b** *CD4* knockout efficiency was determined for the indicated sgRNAs targeting the *CD4* gene, with two versions of the sgRNA structure in Jurkat cells. Cells were analyzed for CD4 expression by flow cytometry 72 hours after transfection. The raw data are shown in Figure S7 in Additional file 1. **c** T→C and T→G mutations are superior to the T→A mutation. Eleven sgRNAs targeting *CCR5* were randomly selected. The knockout efficiency of sgRNAs with different mutations at position 4 in the sequence of continuous Ts were determined as in Fig. 1c. The raw data are shown in Figure S9 in Additional file 1. The graphs represent biological repeats from one of three independent experiments with similar results, shown as mean ± standard deviation (*n* = 3). Significance was calculated using Student's *t*-test: *P < 0.05; **P < 0.01; ***P < 0.005; ****P < 0.001

Based on these results, mutating T→G or C at position 4 and extending the duplex by ~5 bp appears to achieve the optimal sgRNA structure, with the highest knockout efficiency. Therefore, we compared the knockout efficiency of the original and optimized structures for 16 sgRNAs targeting *CCR5*. A typical optimized structure had a T→G mutation at position 4 and extended the duplex by 5 bp. In 15 out of 16 sgRNAs, the optimized structure increased the knockout efficiency significantly and for sp10, 14, 15, 17, and 18 did so dramatically (Fig. 3a; Figure S6 in Additional file 1).

To exclude the possibility that the increase in knockout efficiency using the optimized sgRNA structure is limited to TZM-bl cells or the *CCR5* gene, we also tested eight sgRNAs targeting the *CD4* gene in Jurkat cells. Consistent with the results observed in TZM-bl cells for the *CCR5* gene, the optimized sgRNA design also significantly increased the efficiency of knocking out the *CD4* gene in the Jurkat cell line (Fig. 3b; Figure S7 in Additional file 1). Thus, the optimized sgRNA structure appears to generally increase knockout efficiency.

The beneficial effect of extending the duplex generally reached a peak at around 5 bp of added length (Fig. 2a). To test whether extending the duplex by 5 bp is superior to extending it by 4 bp or 6 bp, we extended the duplex by 4 bp or 6 bp and compared the resulting knockout efficiencies for the 16 sgRNAs in Fig. 3a. As shown in Figure S8 in Additional file 1, extending the duplex by 4 bp or 6 bp appeared to yield similar knockout efficiency as 5 bp in most cases.

Previously, Chen et al. [10] showed that mutating T→A at position 4 in combination with extending the duplex by 5 bp significantly enhanced the imaging efficiency of the dCas9–GFP fusion protein in cells. Our results showed that extending the duplex by 4–6 bp and mutating T→C or G at position 4 significantly increased knockout efficiency. To compare the effect of two sgRNA designs on increasing the knockout efficiency, we randomly selected ten sgRNAs targeting *CCR5* and compared their knockout efficiencies with different mutations. As shown in Fig. 3c, all of the T→C and most (nine out of ten) of the T→G mutations had significantly higher knockout efficiency than the T→A mutation. It is noteworthy that, although in most cases the T→C mutation had a similar level of knockout efficiency as the T→G mutation, it had a significantly higher knockout efficiency in sp11 (+11 %, *P* = 0.006) and sp19 sgRNAs (+6 %, *P* = 0.026) (Fig. 3c; Figure S9 in Additional file 1), suggesting that the T→C mutation might be the best choice.

**Fig. 4:**
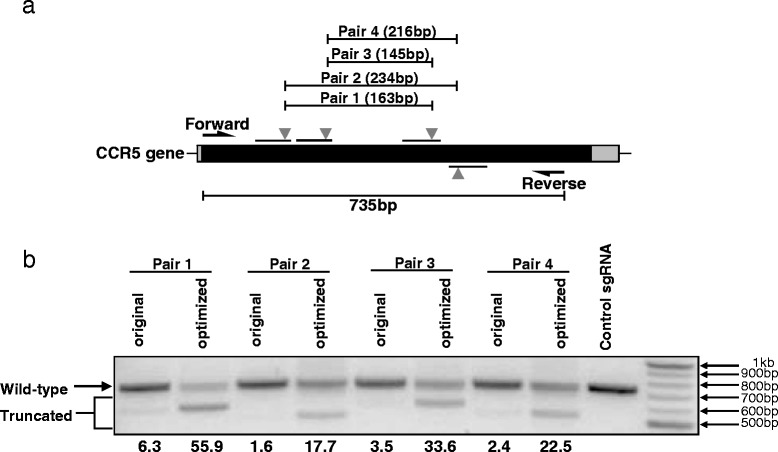
The efficiency of gene deletion is increased dramatically using optimized sgRNAs. **a** The *CCR5* gene deletion. **b** sgRNA pairs targeting *CCR5* with the original or optimized structures were co-transfected into TZM-bl cells with a Cas9-expressing plasmid. The gene deletion efficiency was determined by amplifying the *CCR5* gene fragment. Note that the truncated fragments of CCR5, with a smaller size than wild-type CCR5, are a consequence of gene deletion using paired sgRNAs. The numbers below each lane indicate the percentage deletion

Creation of a frame-shift mutation with an sgRNA is generally insufficient to investigate the loss of function of noncoding genes, such as long noncoding RNAs (lncRNAs) or microRNA genes. A better strategy is to excise all or part of the gene of interest, which requires cutting at two positions simultaneously and linking the two breakpoints together. The efficiency of generating this type of deletion mutation is very low with current sgRNA design templates; however, the deletion efficiency was improved dramatically (around tenfold) in all four pairs of sgRNAs tested here (Fig. 4). If the original sgRNA structure, in which the deletion efficiency ranged from 1.6–6.3 % (Fig. 2c), was used to delete target genes, one would have to screen hundreds of colonies to identify the colonies with the deletion, which is a daunting task. Using the optimized sgRNAs, in which the deletion efficiency ranged from 17.7–55.9 % (Fig. 4), the number of colonies that would need to be screened to identify those with the deletion would be within the limits of feasibility. Thus, the optimized sgRNA template would simplify the genome-editing procedure, thereby enhancing its potential utility.

**Fig. 5:**
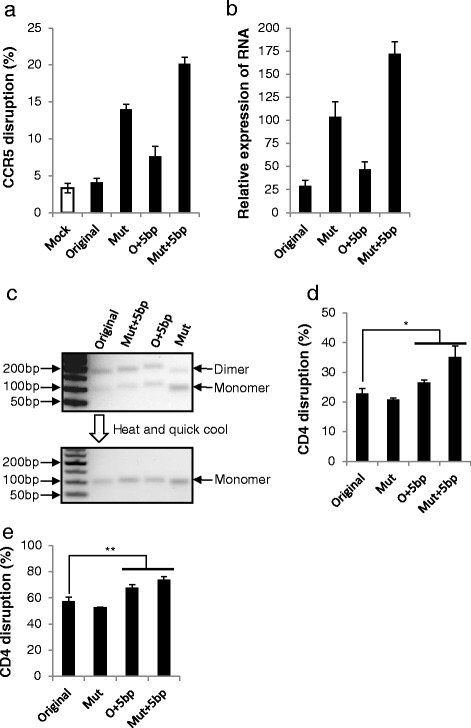
How modifications increase knockout efficiency. **a** Knockout efficiency of sp3 from Fig. 2a with the indicated modifications was determined as in Fig. 1b. The raw data are shown in Figure S10 in Additional file 1. *Mut* mutant, *O* original. **b** sgRNA levels were determined by real-time PCR. The relative expression level was normalized to U6 small RNA. **c** In vitro transcribed sgRNA formed dimers (*upper panel*), which can be transformed into monomers by a heating and quick cooling step (*lower panel*). **d** sp7 from Fig. 3b was transcribed in vitro and preloaded into Cas9. The complex was electroporated into activated primary CD4+ T cells. Knockout efficiency was determined as in Fig. 3b. The raw data are shown in Figure S11 in Additional file 1. **e** In vitro transcribed sp7 was electroporated into TZM-Cas9 cells. Knockout efficiency was determined as in Fig. 3b. The raw data are shown in Figure S11 in Additional file 1. The graphs represent biological repeats from one of three independent experiments with similar results, shown as mean ± standard deviation (*n* = 3). Significance was calculated using Student's *t*-test: *P < 0.05; **P < 0.01

Mutating the contiguous Ts is likely to increase the production of sgRNAs. Thus, to understand how modifications increase the knockout efficiency, we measured the RNA level of different sgRNA structures. First, we checked the *CCR5* knockout efficiency of the sgRNA with the extended duplex or a mutated continuous sequence of Ts or with both. Consistent with our previous study, both modifications individually increased knockout efficiency, and in combination further increased knockout efficiency (Fig. 5a; Figure S10 in Additional file 1). Next, we measured the sgRNA levels in transfected cells. Mutating the continuous sequence of Ts significantly increased the sgRNA level, and it appears that extending the duplex also slightly increased the sgRNA level (Fig. 5b). To ascertain if increased sgRNA production or the sgRNA structure or both is responsible for increased knockout efficacy, we transfected activated CD4+ T cells with Cas9 protein preloaded with in vitro transcribed sgRNAs, which excludes the effect of RNA level change because in this case the amount of sgRNA remains the same. In initial experiments, the results using the in vitro transcribed sgRNAs were highly variable, because these molecules form dimers to variable extents which interfered with their functionality (Fig. 5c). Cas9 can only bind to the monomers but not the dimers, in which the sgRNA structure is not maintained. The ratio of monomers to dimers was not fixed between samples, which led to highly variable results. However, this problem was solved by a heating and quick cooling step (Fig. 5c), as we have previously shown for other small RNAs with duplex structures [12]. With pure monomer sgRNAs, it appeared that Cas9 preloaded with sgRNAs with an extended duplex has higher knockout efficiency (Fig. 5d; Figure S11 in Additional file 1), suggesting that the structural change of extending the duplex can by itself increase Cas9 functionality. Next, we transfected in vitro transcribed sgRNAs into cells stably expressing Cas9 and showed that extending the duplex by itself increases knockout efficiency (Fig. 5e; Figure S11 in Additional file 1), most likely because of the structural change and not because of changes in RNA levels.

**Fig. 6:**
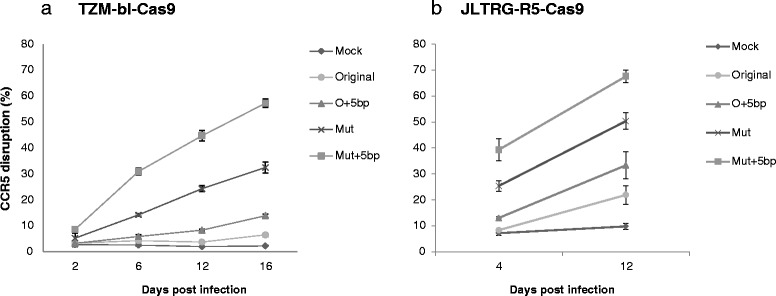
Testing the effect of modifications by lentiviral infection. TZM-bl cells (**a**) or JLTRG-R5 cells (**b**) were infected with Cas9-expressing lentivirus, and cells stably expressing Cas9 were selected. The indicated sgRNA (sp3 from Fig. 2a)-expressing cassettes were packaged into lentivirus and used to infect cells stably expressing Cas9 at MOI = 0.5. Knockout efficiency was determined as in Fig. 1b on the indicated days. The raw data are shown in Figure S12 in Additional file 1. *O* original, *Mut* mutant

We performed all our experiments with transient plasmid transfection, in which the copy number of the Cas9 and the sgRNA can vary considerably. Low multiplicity of infection (MOI) of lentivirus vector harboring the Cas9 or the sgRNA should provide relatively consistent copy numbers of Cas9 and sgRNA in infected cells. Therefore, to determine sgRNA functionality more rigorously, we first created cell lines stably expressing Cas9 by infecting TZM-bl or JLTRG-R5 cells with lentivirus harboring a Cas9-expressing cassette and selecting the cells stably expressing Cas9. We then infected these cells with lentivirus harboring sgRNAs with different structures at low MOI. The results were similar to the experiments done with plasmids in both cell lines. In fact, the difference between structures shown for lentiviral infection was even greater than what we observed with plasmids (Fig. 6; Figure S12 in Additional file 1), suggesting that the optimized sgRNAs are indeed superior to commonly used sgRNA (+85 nucleotides). These results also suggest that the optimized sgRNAs would perform better for CRISPR-Cas9-based genome-wide pooled screenings, which use lentivirus to deliver sgRNAs at low MOI [13–20].

## Discussion

**Fig. 7:**
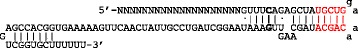
Optimized sgRNA structure. The duplex extension is highlighted in *red*, and the mutation is marked in *bold*. The duplex extension can be four to six nucleotides, and the mutation can be C or G, which showed similar knockout efficiency in most cases

In this study, we systematically investigated the effect of extending the duplex and mutating the continuous sequence of Ts, providing guidance for optimizing sgRNA structure. Our results clearly show that extending the duplex and mutating the continuous sequence of Ts at position 4 to C or G significantly increases knockout efficiency in most cases, and the extent of the improvement in knockout efficiency is striking (Figs. 3 and 4). The general optimized sgRNA structure is illustrated in Fig. 7.

With the optimized structure, most sgRNAs showed high knockout efficiency. Out of a total of 24 sgRNAs with an optimized sgRNA structure tested, 18 showed >50 % knockout efficiency. By contrast, only four sgRNAs showed >50 % knockout efficiency using the original sgRNA structure (Fig. 3a, b). This optimized sgRNA template not only reduces concerns that knockout experiments might not work due to low sgRNA functionality, but also significantly increases the efficiency of more challenging genome-editing procedures, such as gene deletion.

Previously, Hsu et al. [9] showed that extending the duplex by 10 bp in combination with mutating the continuous sequence of Ts did not increase knockout efficiency. Our results show that extending the duplex can significantly increase knockout efficiency, but after reaching a peak at around 5 bp, the effect declines, which might explain this discrepancy. Our conclusion is supported by Chen et al.’s study [10], in which they showed that extending the duplex and mutating the continuous sequence of Ts significantly enhances the imaging efficiency of the dCas9–GFP fusion protein in cells. The effects of these two modifications appear to be different. Mutating the continuous sequence of Ts significantly increased sgRNA production (Fig. 5b), which is likely to be the result of increased transcription efficiency due to the disrupted pause signal [11]. The results with in vitro transcribed sgRNAs suggest that extending the duplex by itself also increases Cas9 functionality because of the structural change (Fig. 5d, e), since any effect of the RNA level was excluded in this experiment. When sgRNA is expressed inside cells, both effects contribute to increase the functionality. It is possible that the modified sgRNA structure might enhance binding to Cas9 or increase its stability. Further work is needed to determine how exactly sgRNA structure increases functionality.

## Conclusions

Extending the duplex by ~5 bp combined with mutating the continuous sequence of Ts at position 4 to C or G significantly increased CRISPR-Cas9 gene knockout efficiency.

## Methods

### Reagents

The TZM-bl cell line (catalog #8129) was obtained from the NIH AIDS Reagent Program and cultured in Dulbecco’s modified Eagle’s medium (DMEM; Life Technologies) with high glucose. The Jurkat (E6-1) cell line (catalog #177) was also obtained from the NIH AIDS Reagent Program and cultured in RPMI medium (Life Technologies). Both media were supplemented with 10 % fetal bovine serum (Life Technologies) and penicillin/streptomycin/L-glutamine (Life Technologies). All cells were maintained at 37 °C and 5 % CO_2_ in a humidified incubator.

Anti-CCR5 antibody (APC-conjugated, catalog #550856, clone 3A9) was purchased from BD Biosciences. Anti-CD4 antibody (APC-conjugated, catalog #317416, clone OKT4) was purchased from Biolegend. Anti-CD4 antibody (FITC-conjugated, catalog #35-0049-T100, clone RPA-T4) was purchased from TONBO Bioscience.

spCas9 protein was custom made (Novoprotein Scientific) and stored at 1 mg/ml concentration in −80 °C.

### Plasmid construction

sgRNA fragments were inserted into pLB vectors (Addgene plasmid #11619) [21] at the Hpa I and Xho I sites. Cloned pLB-sgRNA constructs were sequenced to confirm that the sequence inserted was correct. The oligo sequences are listed in Additional file 3. The sgRNAs were started with either A or G, which is the preferred initiation nucleic acid for the U6 promoter [22]. Plasmids were purified with the EZNA Endo-free Mini-prep kit (Omega Biotech). pSpCas9(BB) (pX330) (catalog #42230) [4] and lentiCas9-Blast (catalog #52962) [17] was purchased from Addgene. pX261-dU6 was constructed from pX261-U6-DR-hEmx1-DR-Cbh-NLS-hSpCas9-NLS-H1-shorttracr-PGK-puro (Addgene plasmid #42337) [4] by deleting a 398-bp fragment by NdeI digestion, followed by Klenow reaction and blunt end ligation to delete part of the U6 expression cassette.

### Determining knockout efficiency

TZM-bl cells (9 × 10^4^ per well) were seeded into 24-well plates overnight before transfection and washed twice with DPBS, and 300 μl of pre-warmed Opti-Mem I medium was added to each well. pLB-sgRNA plasmids (0.5 μg at a concentration of 0.1 μg/ul) were mixed with 0.5 μg of the Cas9 plasmid pX330 pre-mixed in 100 μl of Opti-Mem I medium. Two microliters of Lipofectamine 2000 transfection agent in 100 μl of Opti-Mem I medium per well were added to the diluted plasmids, followed by a 20-minute incubation. The complex was added to the cells, and the medium was changed to complete medium after a 6-hour incubation at 37 °C in 5 % CO_2_. Cells were collected for flow cytometry analysis 48 hours after transfection.

Jurkat cells were transfected with 0.5 μg of the pX330 plasmid and 0.5 μg of pLB-sgRNA constructs using the Neon 10-μl transfection kit (Life Technologies), according to the manufacturer’s instructions, and 2 × 10^5^ cells were used per 10-μl tip. Parameters were set to 1325 V, 10 ms, and three pulses. Cells were collected for flow cytometry analysis 72 hours after transfection.

Cells were stained with either anti-CCR5 antibody for TZM-bl cells or anti-CD4 antibody for Jurkat cells, followed by analysis with a FACScanto II cell analyzer (BD Bioscience). Only GFP-positive cells (GFP is a marker expressed by the pLB vector, serving as positive control for transfection) were analyzed for knockout efficiency.

### Determining the sgRNA expression level

TZM-bl cells (2.5 × 10^5^ per well) were seeded into six-well plates overnight before transfection. Cells were transfected with 1.5 μg of pLB-sgRNA plasmids and 1.5 μg of the Cas9 plasmid pX330 with Lipofectamine 2000 (Life Technologies, catalog #11668019), according to the manufacturer’s instructions. Cells were collected 48 hours after transfection. GFP-positive cells were sorted with a FACSAria II cell sorter (BD Bioscience), followed by small RNA extraction with the miRNeasy Mini kit (Qiagen, catalog #217004). One microgram of extracted RNA was reverse transcribed with SuperScript® III Reverse Transcriptase reaction (Life Technology, catalog #18080-051), according to the manufacturer’s instructions. The cDNAs were quantified with Syber Green qPCR MasterMix (ABI, catalog #4309155) with primers (forward 5’-GTGTTCATCTTTGGTTTTGTGTTT-3’ and reverse 5’-CGGTGCCACTTTTTCAAGTT-3’). U6B was used as the internal control.

### Evaluating target site modification at the DNA level by next-generation sequencing

TZM-bl cells were transfected with Lipofectamine 2000 in six-well plates, according to the manufacturer’s instructions. Cells were collected 48 hours after transfection. GFP-positive cells were sorted using a FACSAria II cell sorter (BD Bioscience), followed by genomic DNA extraction with the QIAamp DNA Blood Mini kit. *CCR5* gene fragments were amplified with the primers CCR5-DS-F (5’-ACACTCTTTCCCTACACGACGCTCTTCCGATCTTCTACCTGCTCAACCTGGCC-3’) and CCR5-DS-R (5’-GTGACTGGAGTTCAGACGTGTGCTCTTCCGATCAAGTCCCACTGGGCGGC-3’). The resulting PCR products were amplified for a second round of PCR with individual index primers. The amplicons were run on a 2.5 % agarose gel and purified with the QIAquick Gel Extraction kit (QIAGEN, catalog #28704). Equal amounts of amplicons were mixed and sequenced with a MiSeq sequencer (Illumina).

### Evaluating *CCR5* disruption efficiency with lentiviral delivery of sgRNA

Lenti-Cas9-Blast and the Viral Power packaging mix (Life Technology, catalog #K4975-00) were co-transfected into 293 T cells with the calcium phosphate transfection protocol. Supernatant was collected and filtered through a 0.45-μm filter before being used for infection of TZM-bl cells and JLTRG-R5 cells (NIH AIDS Reagent Program #11586). Cells (2 × 10^6^) were seeded into a 10-cm dish. After overnight culture, cells were infected with 1 ml viral supernatant with 5 ng/ml polybrene for 3 hours. Forty-eight hours after infection, the cells were treated with 10 μg/ml blasticidin (Life Technology, catalog #R210-01) for 3 days. The surviving cells were labeled as TZM-Cas9 or JLTRG-R5-Cas9 cells.

pLB-sgRNAs were packaged into lentivirus in a similar manner as Lenti-Cas9-Blast. TZM-Cas9 or JLTRG-R5-Cas9 cells (1 × 10^5^) were seeded into 24-well plates and infected at MOI = 0.5. A portion of the cells were collected at different time points and analyzed by FACS to determine the *CCR5* disruption rate. The rate of occurrence of GFP-positive cells was ~30 % for TZM-bl-Cas9 cells or ~10 % for JLTRG-R5-Cas9 cells.

### Knockout of *CD4* in primary CD4+ T cells with Cas9 preloaded with in vitro transcribed sgRNA

CD4+ T cells were isolated from peripheral blood mononuclear cells with StemSep™ Human CD4+ T Cell Enrichment Kit (StemCell Technologies, catalog #14052), and activated with Dynabeads® Human T-Activator CD3/CD28 (Life Technology, catalog #11131D) for 5 days in the presence of 20 U/ml IL-2 (NIH AIDS Reagents Program, catalog #136), 10 % fetal calf serum, and 1× penicillin-streptomycin-glutamine solution (Life Technology, catalog #10378-016).

sgRNAs were transcribed with HiScribe T7High Yield RNA Synthesis kit (NEB) according to the manufacturer’s instructions, followed by purification with the RNeasy Mini kit (Qiagen, catalog #217004). Before each use, sgRNAs were heated to 95 °C for 3 minutes in a PCR tube and immediately transferred to a water/ice bath for 2 minutes to obtain pure monomers.

Activated primary CD4+ T cells were electroporated using the Neon transfection system (100 μl tip, Life Technologies, catalog #MPK10096) with 10 μg of spCas9 protein that was preloaded with 300 pmol sgRNA (mixed and incubated at room temperature for 10 minutes). Cells (1 × 10^6^) resuspended in 100 μl R buffer were mixed with a protein:RNA mix, followed by Neon electroporation (1500 V, 10 ms, three pulses), according to the manufacturer’s instructions. After 48 hours, the cells were stained with CD4 antibody and subjected to FACS analysis.

TZM-Cas9 cells were electroporated by Neon transfection system (10 μl tip; Life Technology catalog #MPK1096) with 30 pmol sgRNA. Cells (5 × 10^4^) were re-suspended in 10 μl R buffer and mixed with RNA, followed by Neon electroporation (1005 V, 35 ms, two pulses) according to the manufacturer’s instructions. After 48 hours, the cells were stained with CD4 antibody and subject to FACS analysis.

### Gene deletion assay

TZM-bl cells were co-transfected with sgRNA pairs (0.25 μg each) along with 0.5 μg of the Cas9-expressing plasmid pX261-dU6.sgRNA: pair 1 was *CCR5* sp7 plus sp14; pair 2 was *CCR5* sp7 plus sp18; pair 3 was *CCR5* sp10 plus sp14; and pair 4 was *CR5* sp10 plus sp18. The sgRNA sequences are provided in Additional file 3. Twenty-four hours after transfection, the cells were treated with 0.8 μg/ml puromycin for 48 hours, followed by recovery in medium without puromycin for 5 days. Genomic DNA was extracted from cells with the GenElute™ Mammalian Genomic DNA Miniprep kit (Sigma-Aldrich, catalog #G1N70). *CCR5* gene fragments were amplified from 70 μg of genomic DNA using Premix Ex Taq (Takara, catalog #RR003A) with forward primer 5’-ATGGATTATCAAGTGTCAAGTCCAA-3’ and reverse primer 5’-AGGGAGCCCAGAAGAGAAAATAAAC-3’ for the *CCR5* gene. The PCR was stopped at different cycle numbers to check the amount of amplicon and ensure that the amplification was in the exponential phase. PCR amplicons were analyzed on a 1 % agarose gel.

### Statistical analysis

Student’s *t*-test (two-tailed, assuming equal variances for all experimental data sets) was used to compare two groups of independent samples.

### Data availability

The data set supporting the results of Fig. 1b in this article is available in the Gene Expression Omnibus with accession code GSE74766 (http://www.ncbi.nlm.nih.gov/geo/query/acc.cgi?acc=GSE74766).

## Additional files

Additional file 1: Figure S1.
**a** Native crRNA-tracrRNA duplex. **b** Short sgRNA (+48 nucleotides). **c** Commonly used lone sgRNA (+85 nucleotides). **Figure S2.** *CCR5* knockout efficiency as determined by flow cytometry for the sgRNAs in Fig. 1b, c. **Figure S3.** *CCR5* knockout efficiency as determined by flow cytometry for the sgRNAs in Fig. 1d. **Figure S4.** *CCR5* knockout efficiency as determined by flow cytometry for the sgRNAs in Fig. 2a. **Figure S5.** *CCR5* knockout efficiency as determined by flow cytometry for the sgRNAs in Fig. 2b. **Figure S6.** *CCR5* knockout efficiency as determined by flow cytometry for the sgRNAs in Fig. 3a. **Figure S7.** *CD4* knockout efficiency as determined by flow cytometry for the sgRNAs in Fig. 3b. **Figure S8.** *CCR5* knockout efficiency for the indicated sgRNAs with a 4-, 5-, or 6-bp duplex extension. CCR5 expression was determined in the same way as in Fig. 1b. **Figure S9.** CCR5 knockout efficiency for the indicated sgRNAs with T→A, T→C and T→G mutations for the sgRNAs in Fig. 3c. **Figure S10.** CCR5 knockout efficiency as determined by flow cytometry for the sgRNAs in Fig. 5a. **Figure S11.** CD4 knockout efficiency as determined by flow cytometry for the sgRNAs in Fig. 5d, e. **Figure S12.** CCR5 knockout efficiency as determined by flow cytometry for the sgRNAs in Fig. 6. (PDF 2487 kb) Additional file 2:
**Dataset S1.** Deep sequencing raw data of CCR5 target site modification of Fig. 2b. (XLSX 236 kb) Additional file 3: Table S1. sgRNA sequences. (XLSX 14 kb)

## Abbreviations

bp: Base pair
CRISPR: Clustered regularly interspaced short palindromic repeat
crRNA: CRISPR RNA
FACS: Fluorescence-activated cell sorting
GFP: Green fluorescent protein
MOI: Multiplicity of infection
PCR: Polymerase chain reaction
sgRNA: Single-guide RNA
tracrRNA: Trans-activating crRNA
