# Supplementary material for: Optimizing sgRNA structure to improve CRISPR-Cas9 knockout efficiency
Source: Genome Biol. 2015 Dec 15;16:280. doi: 10.1186/s13059-015-0846-3 (PMC4699467; doi:10.1186/s13059-015-0846-3)
Supplement: Additional file 1: Figure S1. — a Native crRNA-tracrRNA duplex. b Short sgRNA (+48 nucleotides). c Commonly used lone sgRNA (+85 nucleotides). Figure S2. CCR5 knockout efficiency as determined by flow cytometry for the sgRNAs in Fig. 1b, c. Figure S3. CCR5 knockout efficiency as determined by flow cytometry for the sgRNAs in Fig. 1d. Figure S4. CCR5 knockout efficiency as determined by flow cytometry for the sgRNAs in Fig. 2a. Figure S5. CCR5 knockout efficiency as determined by flow cytometry for the sgRNAs in Fig. 2b. Figure S6. CCR5 knockout efficiency as determined by flow cytometry for the sgRNAs in Fig. 3a. Figure S7. CD4 knockout efficiency as determined by flow cytometry for the sgRNAs in Fig. 3b. Figure S8. CCR5 knockout efficiency for the indicated sgRNAs with a 4-, 5-, or 6-bp duplex extension. CCR5 expression was determined in the same way as in Fig. 1b. Figure S9. CCR5 knockout efficiency for the indicated sgRNAs with T→A, T→C and T→G mutations for the sgRNAs in Fig. 3c. Figure S10. CCR5 knockout efficiency as determined by flow cytometry for the sgRNAs in Fig. 5a. Figure S11. CD4 knockout efficiency as determined by flow cytometry for the sgRNAs in Fig. 5d, e. Figure S12. CCR5 knockout efficiency as determined by flow cytometry for the sgRNAs in Fig. 6. (PDF 2487 kb) [file 13059_2015_846_MOESM1_ESM.pdf]

Figure S1

a

```
                5’-NNNNNNNNNNNNNNNNNNNNGUUUUAGAGCUAUGCUGUUUUG-3’
                                       ·|||||· ||||||||||||||
 AGCCACGGUGAAAAAGUUCAACUAUUGCCUGAUCGGAAUAAAAUU CGAUACGACAAAACUUACCAAGG-5’
G |||||||                                    GAA
 UCGGUGCUUUUUUU-3’
```

b

```
                                                   g
                5’-NNNNNNNNNNNNNNNNNNNNGUUUUAGAGCUA a
                                       ·|||||· ||||
                        3’-GCCUGAUCGGAAUAAAAUU CGAU a
                                             GAA   a
```

c

```
                                                   g
                5’-NNNNNNNNNNNNNNNNNNNNGUUUUAGAGCUA a
                                       ·|||||· ||||
 AGCCACGGUGAAAAAGUUCAACUAUUGCCUGAUCGGAAUAAAAUU CGAU a
G |||||||                                    GAA   a
 UCGGUGCUUUUU-3’
```

Figure S2

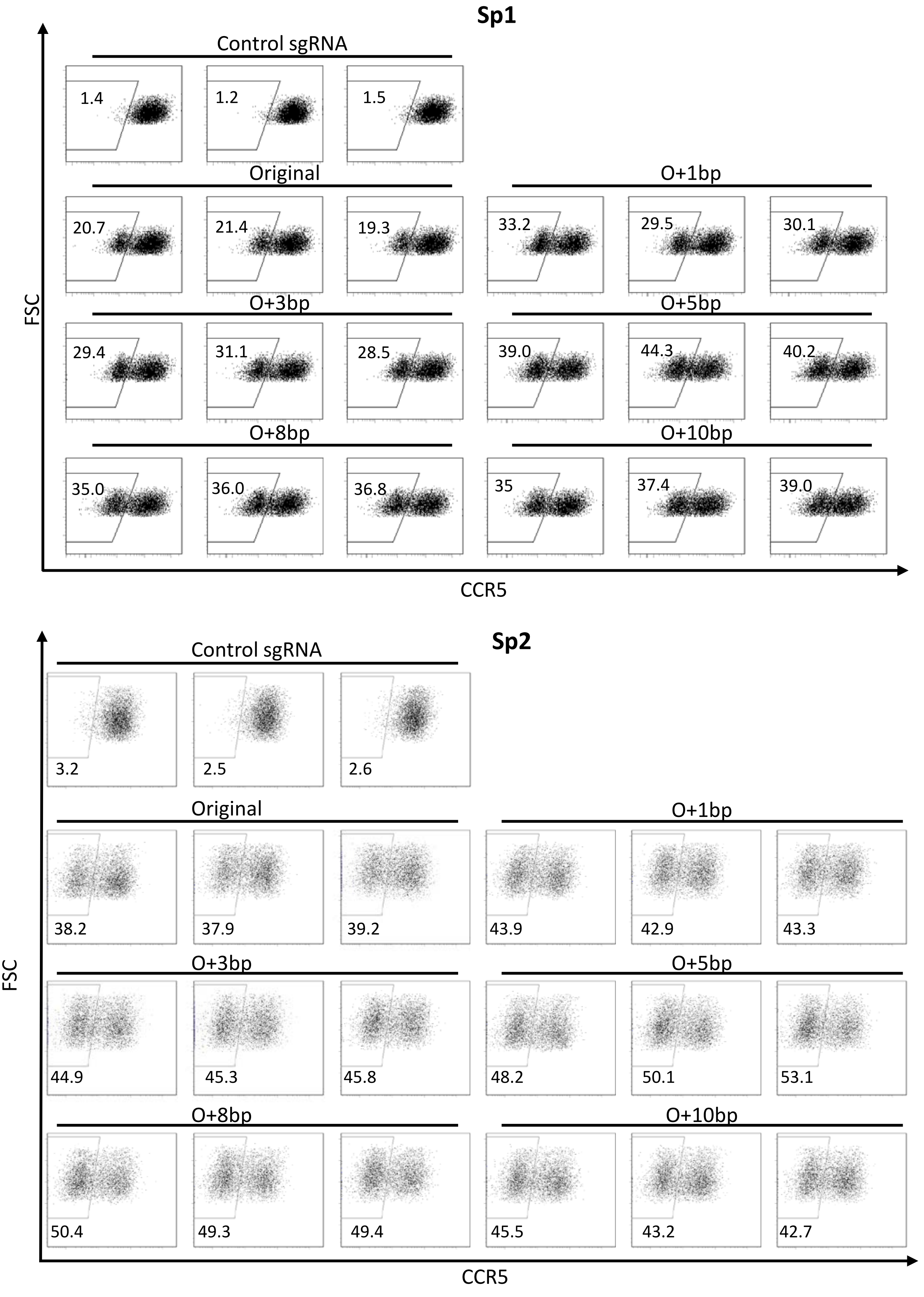
Sp1
Control sgRNA
1.4
1.2
1.5
Original
20.7
21.4
19.3
O+1bp
33.2
29.5
30.1
O+3bp
29.4
31.1
28.5
O+5bp
39.0
44.3
40.2
O+8bp
35.0
36.0
36.8
O+10bp
35
37.4
39.0
FSC
CCR5
Sp2
Control sgRNA
3.2
2.5
2.6
Original
38.2
37.9
39.2
O+1bp
43.9
42.9
43.3
O+3bp
44.9
45.3
45.8
O+5bp
48.2
50.1
53.1
O+8bp
50.4
49.3
49.4
O+10bp
45.5
43.2
42.7
FSC
CCR5

Figure S3

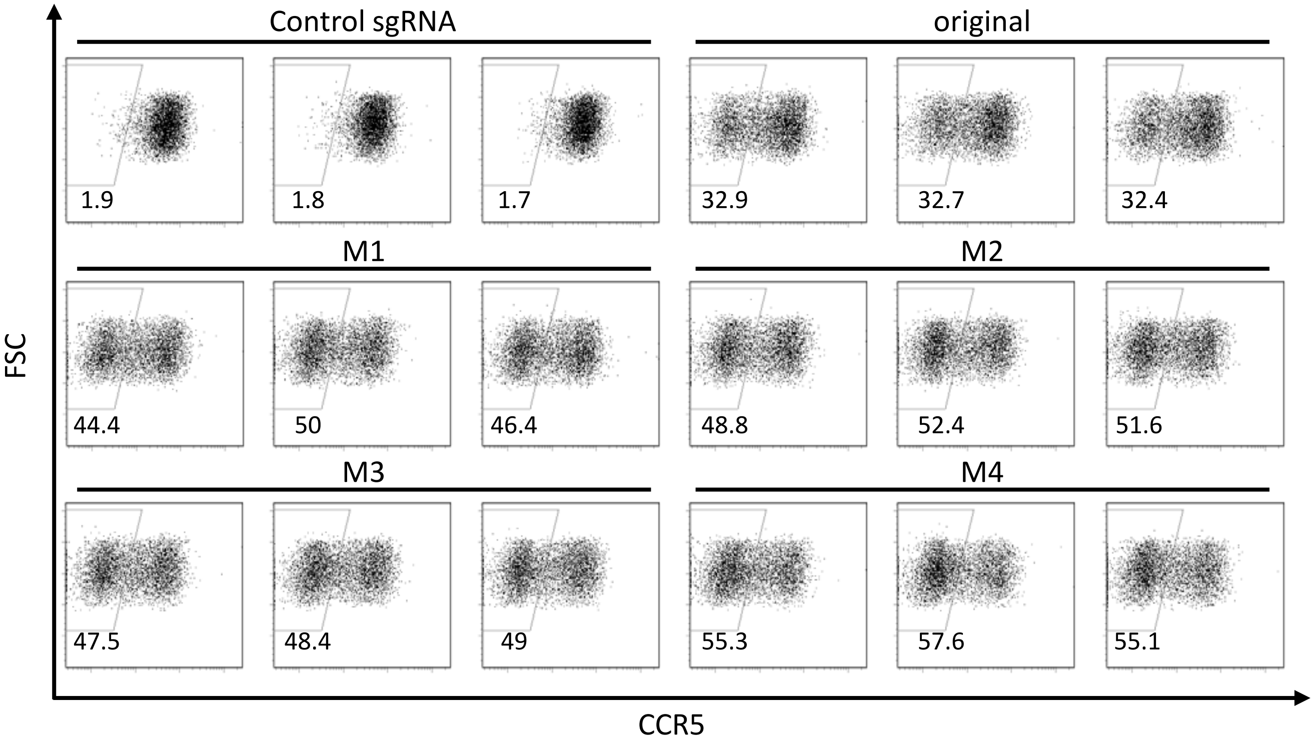
Control sgRNA
original
1.9
1.8
1.7
32.9
32.7
32.4
M1
M2
44.4
50
46.4
48.8
52.4
51.6
M3
M4
47.5
48.4
49
55.3
57.6
55.1
FSC
CCR5

Figure S4

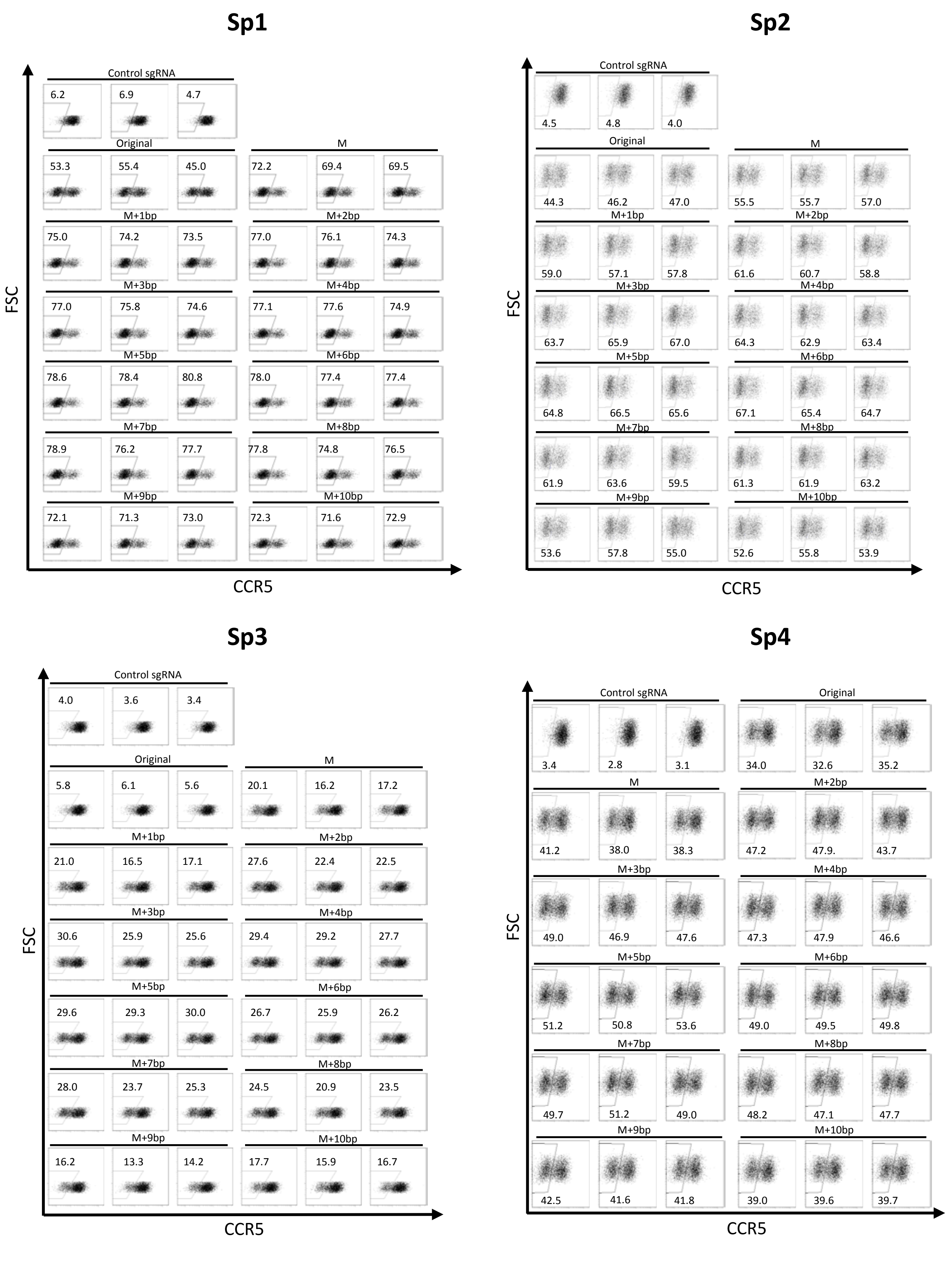
Sp1
Control sgRNA
6.2 6.9 4.7
Original
53.3 55.4 45.0
M
72.2 69.4 69.5
M+1bp
75.0 74.2 73.5
M+2bp
77.0 76.1 74.3
M+3bp
77.0 75.8 74.6
M+4bp
77.1 77.6 74.9
M+5bp
78.6 78.4 80.8
M+6bp
78.0 77.4 77.4
M+7bp
78.9 76.2 77.7
M+8bp
77.8 74.8 76.5
M+9bp
72.1 71.3 73.0
M+10bp
72.3 71.6 72.9
FSC
CCR5
Sp2
Control sgRNA
4.5 4.8 4.0
Original
44.3 46.2 47.0
M
55.5 55.7 57.0
M+1bp
59.0 57.1 57.8
M+2bp
61.6 60.7 58.8
M+3bp
63.7 65.9 67.0
M+4bp
64.3 62.9 63.4
M+5bp
64.8 66.5 65.6
M+6bp
67.1 65.4 64.7
M+7bp
61.9 63.6 59.5
M+8bp
61.3 61.9 63.2
M+9bp
53.6 57.8 55.0
M+10bp
52.6 55.8 53.9
FSC
CCR5
Sp3
Control sgRNA
4.0 3.6 3.4
Original
5.8 6.1 5.6
M
20.1 16.2 17.2
M+1bp
21.0 16.5 17.1
M+2bp
27.6 22.4 22.5
M+3bp
30.6 25.9 25.6
M+4bp
29.4 29.2 27.7
M+5bp
29.6 29.3 30.0
M+6bp
26.7 25.9 26.2
M+7bp
28.0 23.7 25.3
M+8bp
24.5 20.9 23.5
M+9bp
16.2 13.3 14.2
M+10bp
17.7 15.9 16.7
FSC
CCR5
Sp4
Control sgRNA
3.4 2.8 3.1
Original
34.0 32.6 35.2
M
41.2 38.0 38.3
M+2bp
47.2 47.9. 43.7
M+3bp
49.0 46.9 47.6
M+4bp
47.3 47.9 46.6
M+5bp
51.2 50.8 53.6
M+6bp
49.0 49.5 49.8
M+7bp
49.7 51.2 49.0
M+8bp
48.2 47.1 47.7
M+9bp
42.5 41.6 41.8
M+10bp
39.0 39.6 39.7
FSC
CCR5

Figure S5

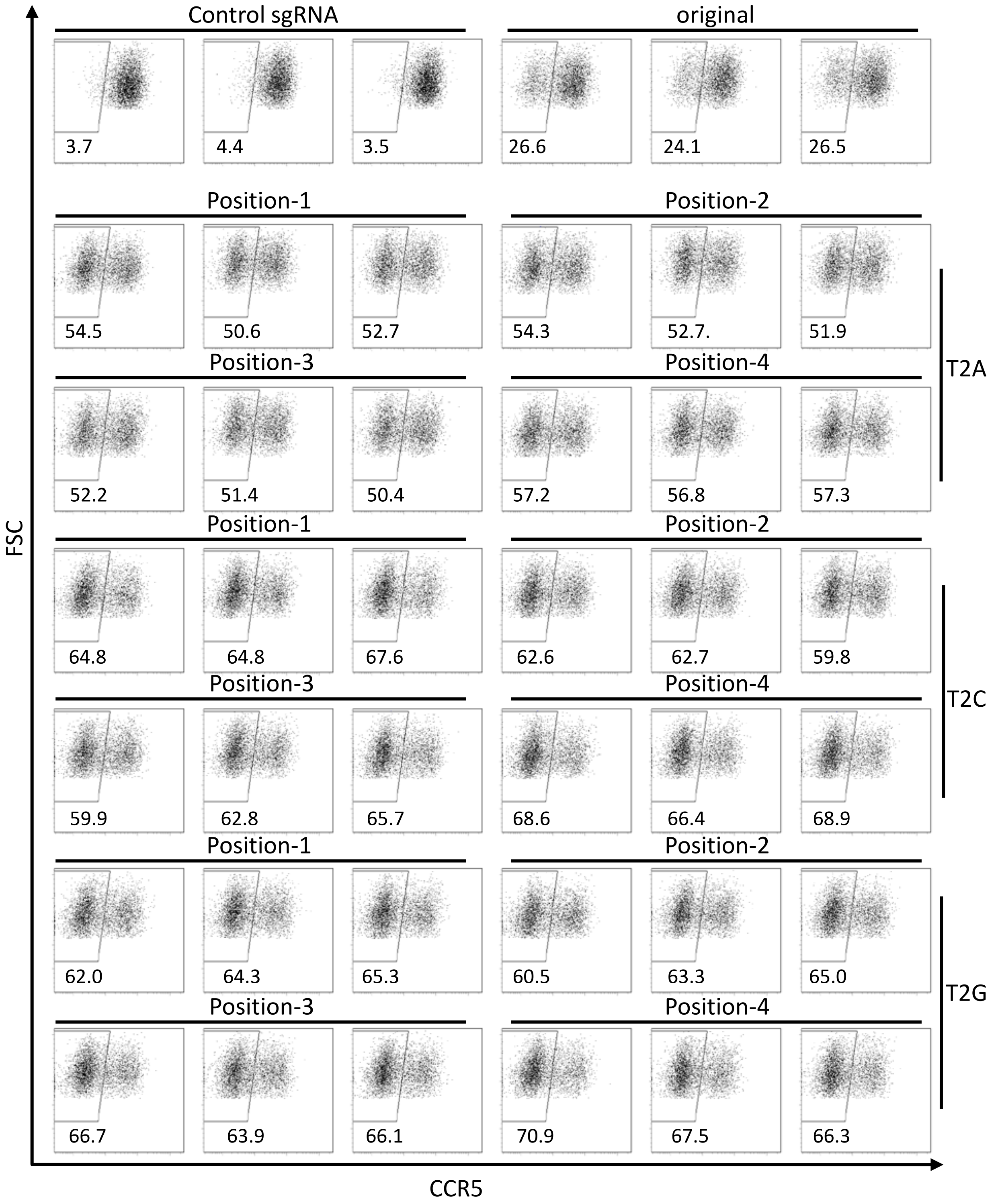
Control sgRNA
original
3.7
4.4
3.5
26.6
24.1
26.5
Position-1
Position-2
54.5
50.6
52.7
54.3
52.7.
51.9
Position-3
Position-4
52.2
51.4
50.4
57.2
56.8
57.3
T2A
Position-1
Position-2
64.8
64.8
67.6
62.6
62.7
59.8
Position-3
Position-4
59.9
62.8
65.7
68.6
66.4
68.9
T2C
Position-1
Position-2
62.0
64.3
65.3
60.5
63.3
65.0
Position-3
Position-4
66.7
63.9
66.1
70.9
67.5
66.3
T2G
FSC
CCR5

Figure S6

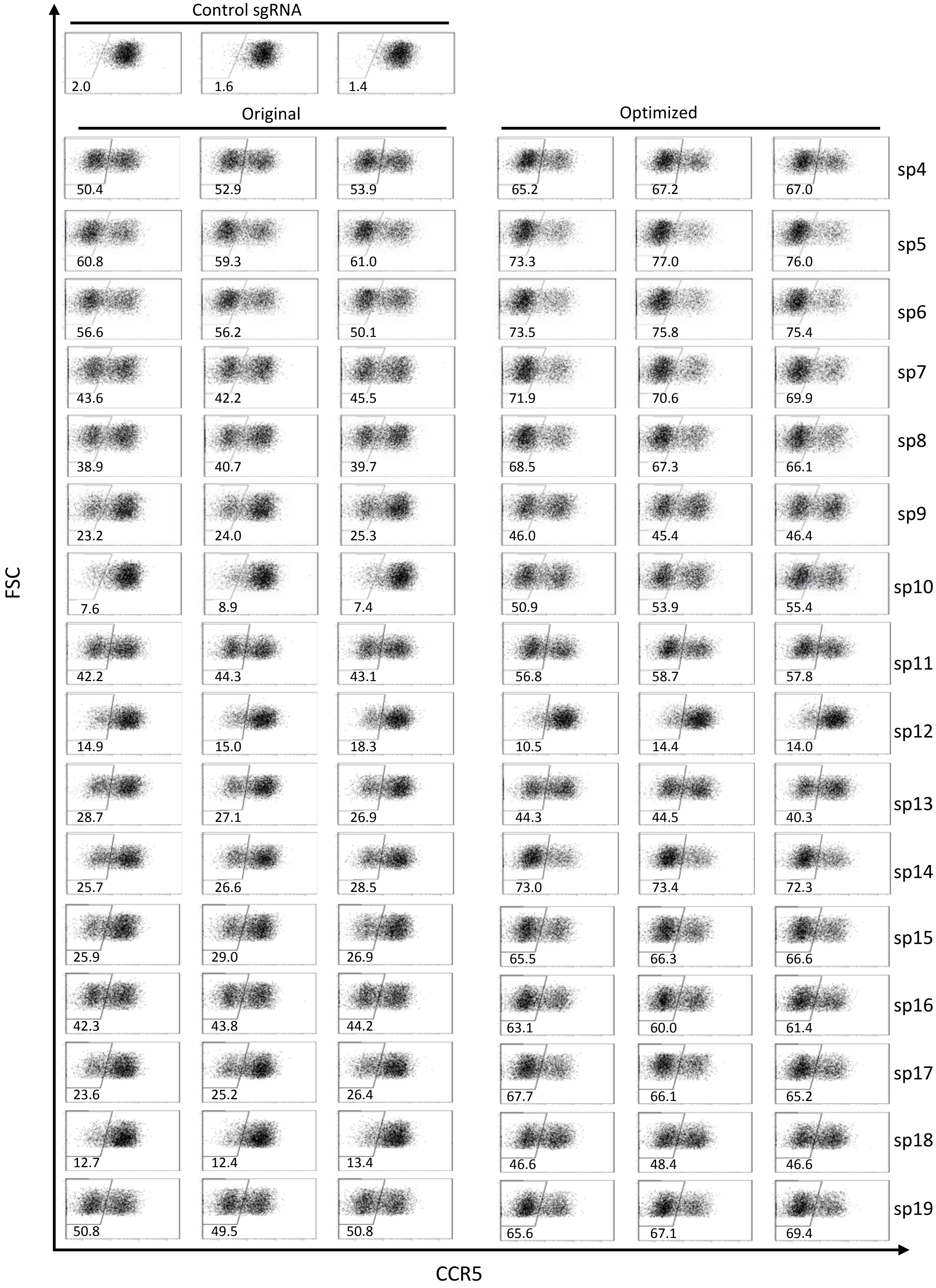

Control sgRNA
2.0
1.6
1.4
Original
Optimized
sp4
50.4
52.9
53.9
65.2
67.2
67.0
sp5
60.8
59.3
61.0
73.3
77.0
76.0
sp6
56.6
56.2
50.1
73.5
75.8
75.4
sp7
43.6
42.2
45.5
71.9
70.6
69.9
sp8
38.9
40.7
39.7
68.5
67.3
66.1
sp9
23.2
24.0
25.3
46.0
45.4
46.4
sp10
7.6
8.9
7.4
50.9
53.9
55.4
sp11
42.2
44.3
43.1
56.8
58.7
57.8
sp12
14.9
15.0
18.3
10.5
14.4
14.0
sp13
28.7
27.1
26.9
44.3
44.5
40.3
sp14
25.7
26.6
28.5
73.0
73.4
72.3
sp15
25.9
29.0
26.9
65.5
66.3
66.6
sp16
42.3
43.8
44.2
63.1
60.0
61.4
sp17
23.6
25.2
26.4
67.7
66.1
65.2
sp18
12.7
12.4
13.4
46.6
48.4
46.6
sp19
50.8
49.5
50.8
65.6
67.1
69.4
FSC
CCR5

Figure S7

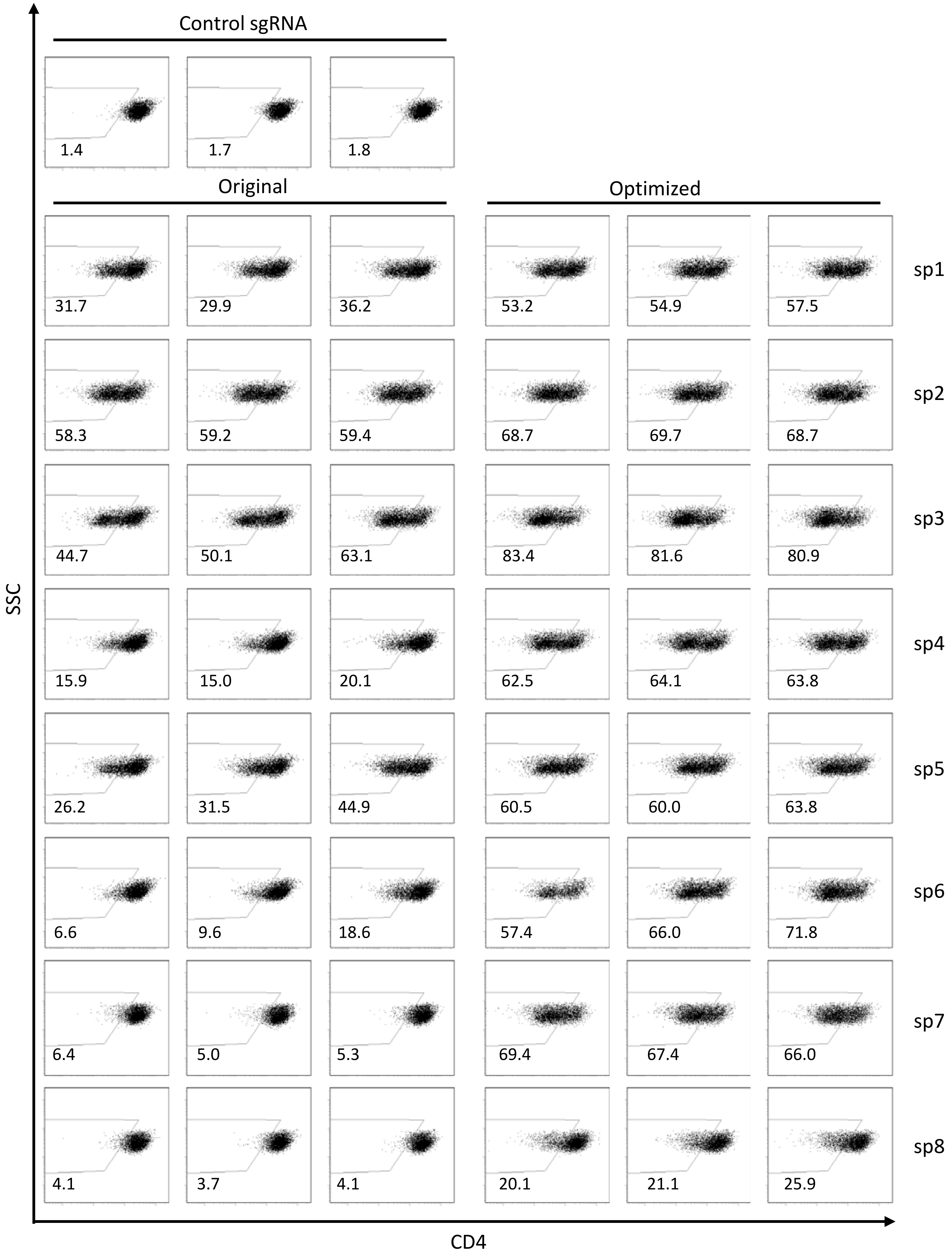
Control sgRNA
1.4
1.7
1.8
Original
Optimized
31.7
29.9
36.2
53.2
54.9
57.5
sp1
58.3
59.2
59.4
68.7
69.7
68.7
sp2
44.7
50.1
63.1
83.4
81.6
80.9
sp3
15.9
15.0
20.1
62.5
64.1
63.8
sp4
26.2
31.5
44.9
60.5
60.0
63.8
sp5
6.6
9.6
18.6
57.4
66.0
71.8
sp6
6.4
5.0
5.3
69.4
67.4
66.0
sp7
4.1
3.7
4.1
20.1
21.1
25.9
sp8
SSC
CD4

Figure S8

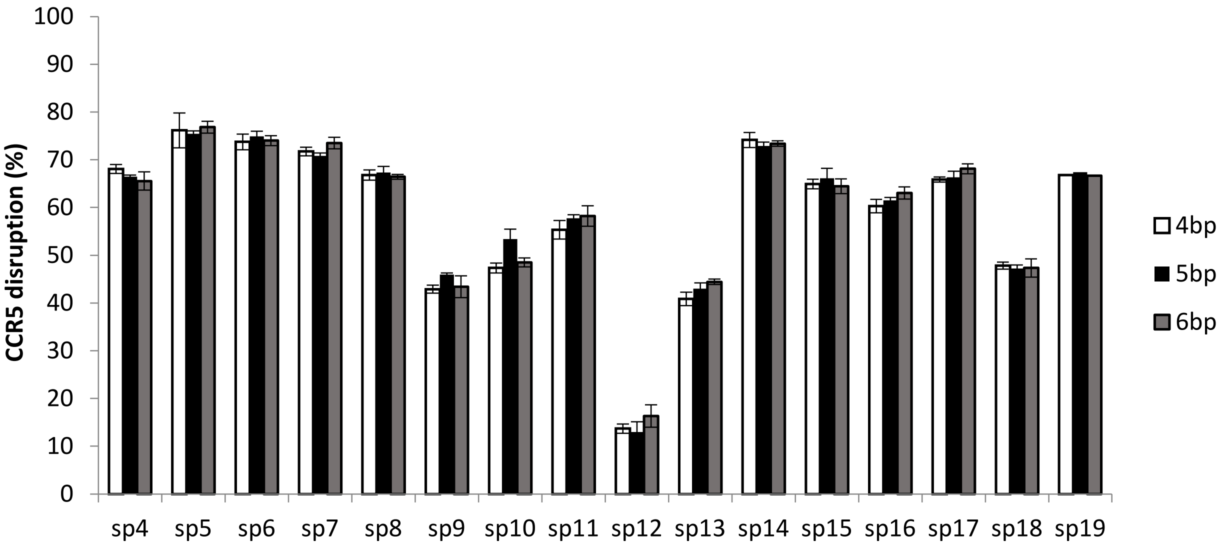

100
90
80
70
60
50
40
30
20
10
0
CCR5 disruption (%)
sp4
sp5
sp6
sp7
sp8
sp9
sp10
sp11
sp12
sp13
sp14
sp15
sp16
sp17
sp18
sp19
4bp
5bp
6bp

Figure S9

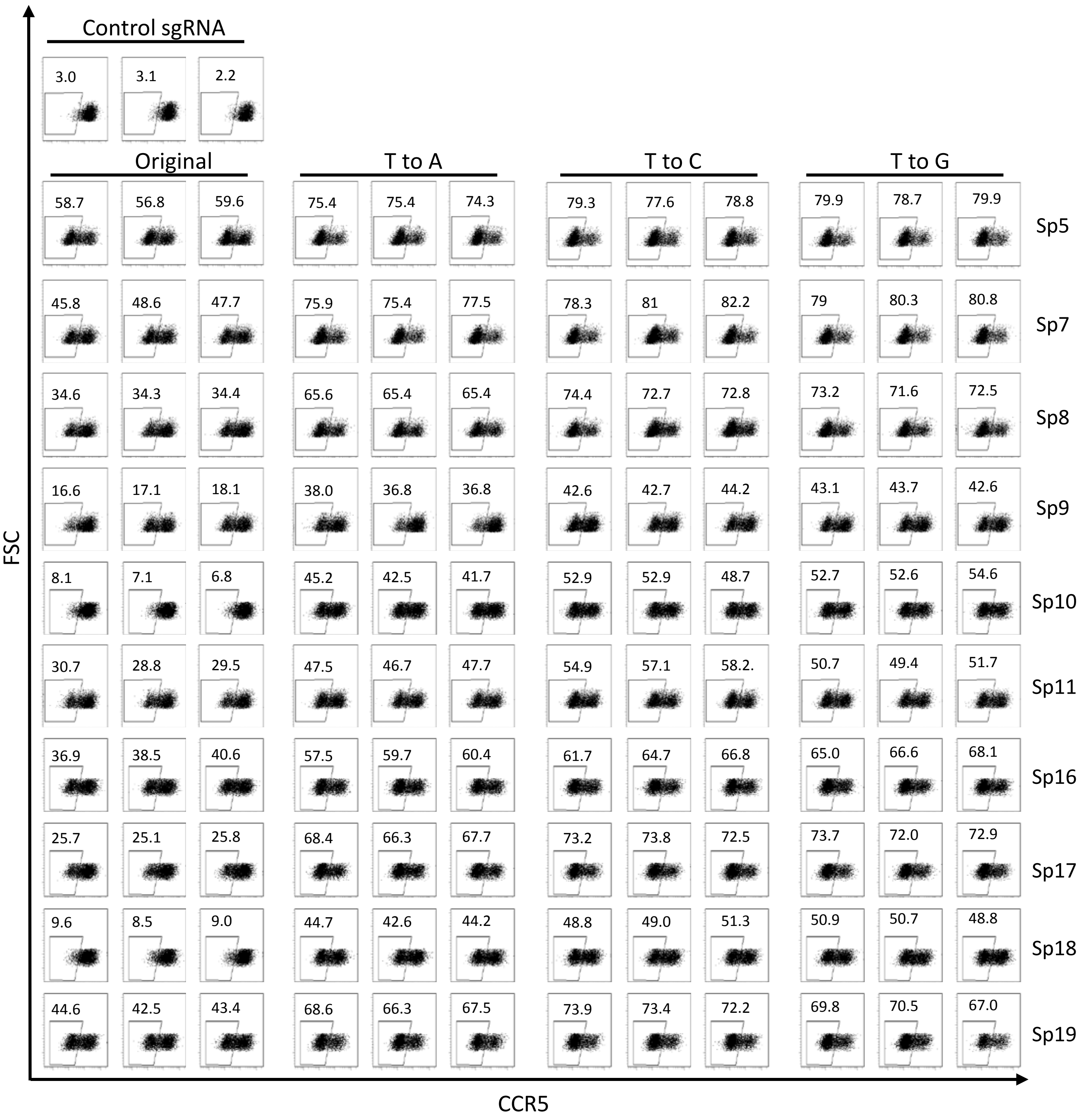
Control sgRNA
3.0
3.1
2.2
Original
T to A
T to C
T to G
58.7 56.8 59.6 75.4 75.4 74.3 79.3 77.6 78.8 79.9 78.7 79.9 Sp5
45.8 48.6 47.7 75.9 75.4 77.5 78.3 81 82.2 79 80.3 80.8 Sp7
34.6 34.3 34.4 65.6 65.4 65.4 74.4 72.7 72.8 73.2 71.6 72.5 Sp8
16.6 17.1 18.1 38.0 36.8 36.8 42.6 42.7 44.2 43.1 43.7 42.6 Sp9
8.1 7.1 6.8 45.2 42.5 41.7 52.9 52.9 48.7 52.7 52.6 54.6 Sp10
30.7 28.8 29.5 47.5 46.7 47.7 54.9 57.1 58.2. 50.7 49.4 51.7 Sp11
36.9 38.5 40.6 57.5 59.7 60.4 61.7 64.7 66.8 65.0 66.6 68.1 Sp16
25.7 25.1 25.8 68.4 66.3 67.7 73.2 73.8 72.5 73.7 72.0 72.9 Sp17
9.6 8.5 9.0 44.7 42.6 44.2 48.8 49.0 51.3 50.9 50.7 48.8 Sp18
44.6 42.5 43.4 68.6 66.3 67.5 73.9 73.4 72.2 69.8 70.5 67.0 Sp19
FSC
CCR5

Figure S10

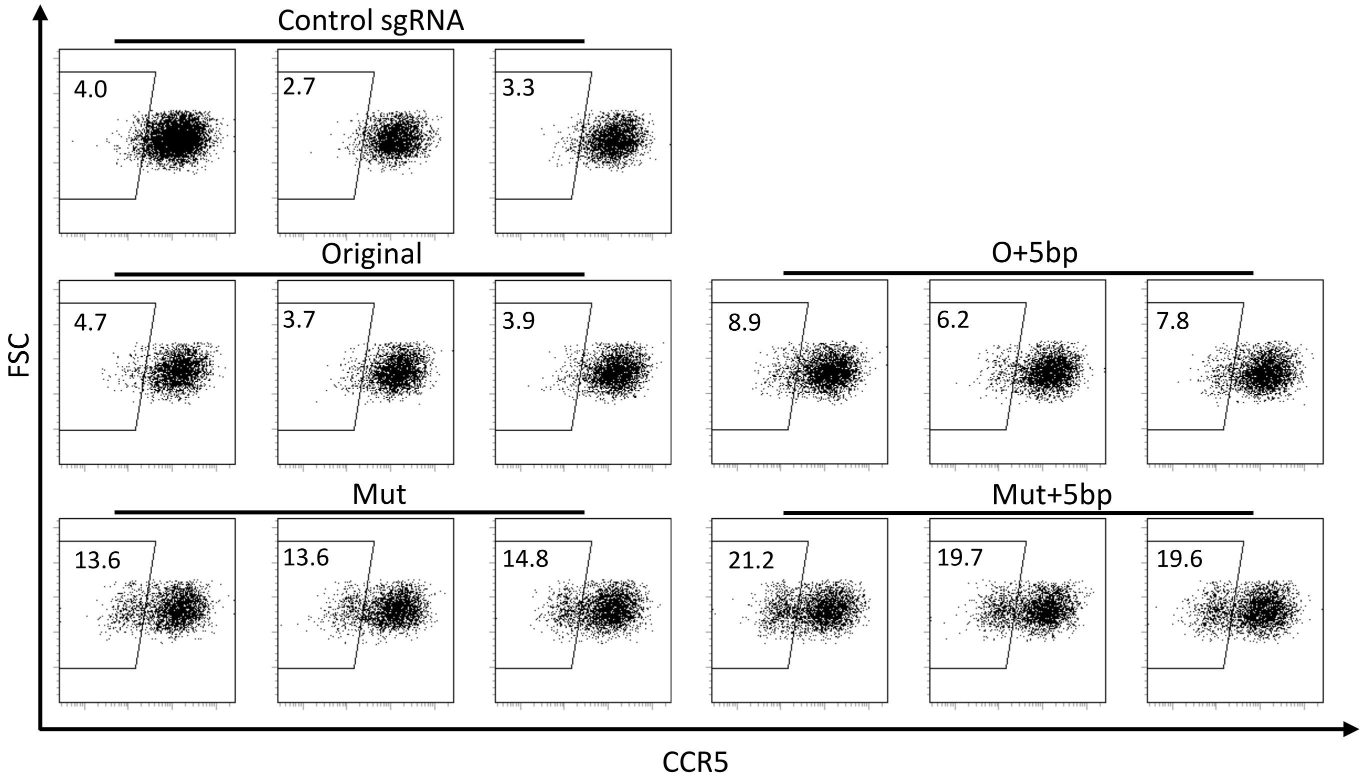
Control sgRNA
4.0
2.7
3.3
Original
4.7
3.7
3.9
O+5bp
8.9
6.2
7.8
Mut
13.6
13.6
14.8
Mut+5bp
21.2
19.7
19.6
FSC
CCR5

Figure S11

Figure 5d raw data

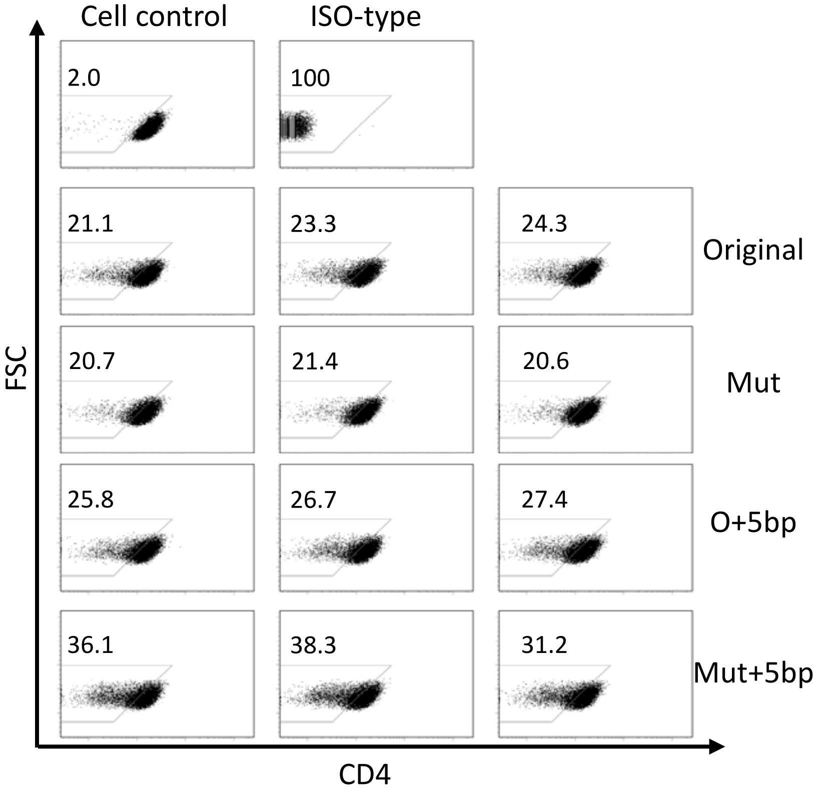

Figure 5e raw data

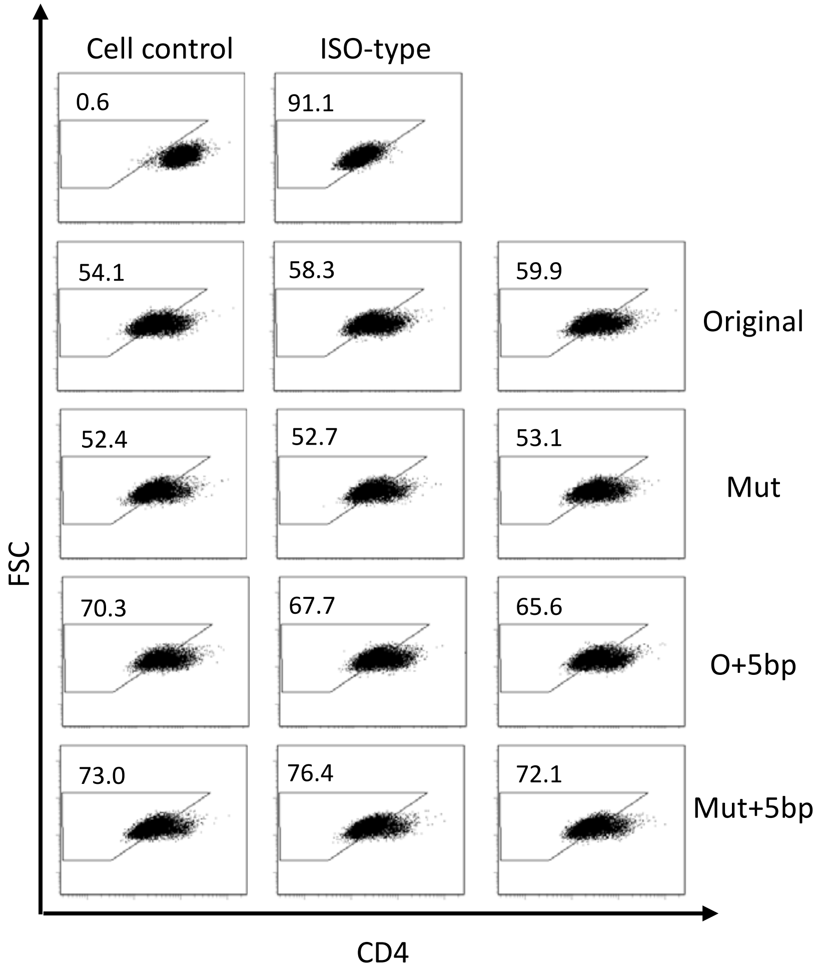

Figure S12

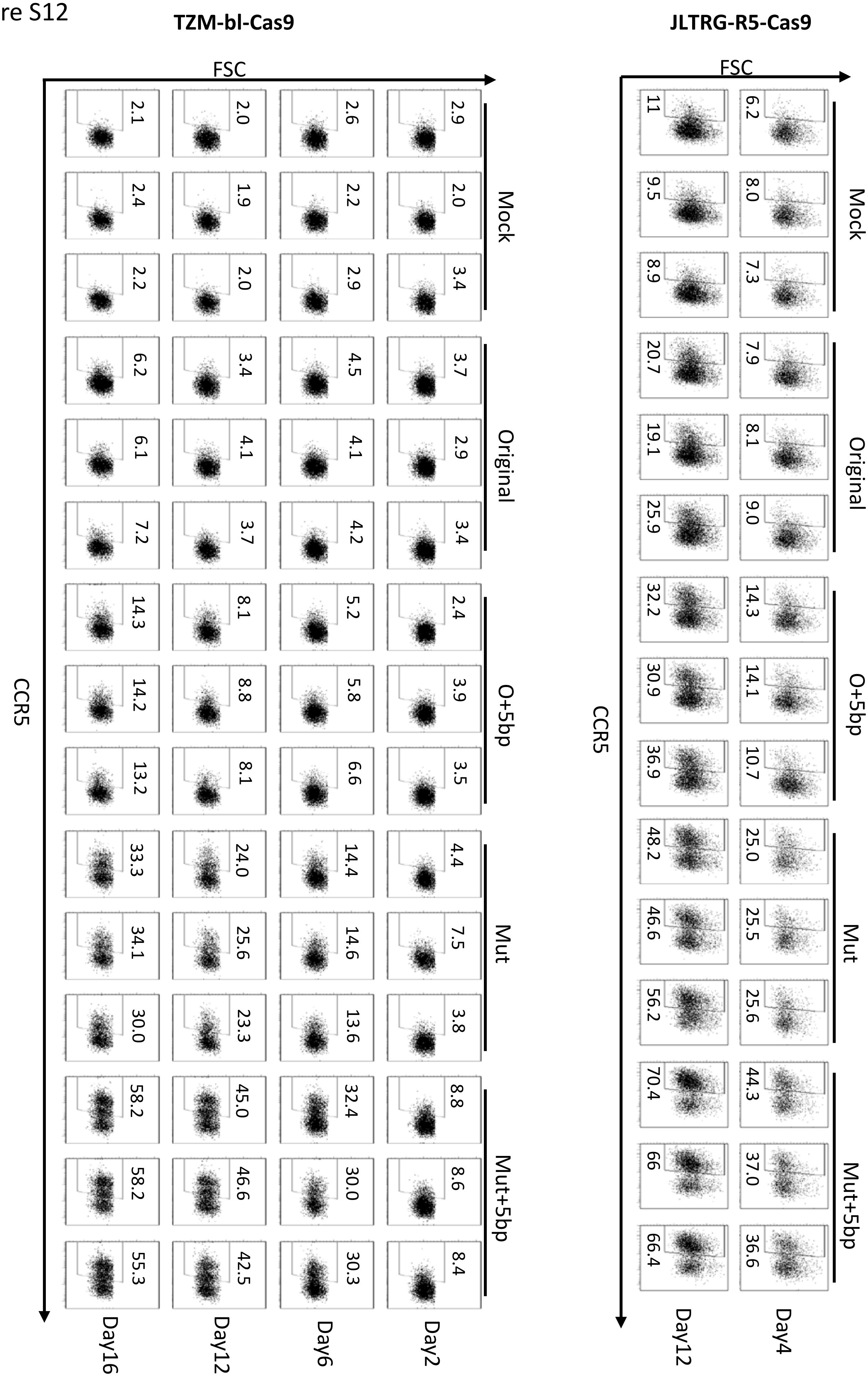
TZM-bl-Cas9
JLTRG-R5-Cas9
FSC
CCR5
Mock
Original
O+5bp
Mut
Mut+5bp
Day2
2.9 2.0 3.4 3.7 2.9 3.4 2.4 3.9 3.5 4.4 7.5 3.8 8.8 8.6 8.4
Day6
2.6 2.2 2.9 4.5 4.1 4.2 5.2 5.8 6.6 14.4 14.6 13.6 32.4 30.0 30.3
Day12
2.0 1.9 2.0 3.4 4.1 3.7 8.1 8.8 8.1 24.0 25.6 23.3 45.0 46.6 42.5
Day16
2.1 2.4 2.2 6.2 6.1 7.2 14.3 14.2 13.2 33.3 34.1 30.0 58.2 58.2 55.3
Day4
6.2 8.0 7.3 7.9 8.1 9.0 14.3 14.1 10.7 25.0 25.5 25.6 44.3 37.0 36.6
Day12
11 9.5 8.9 20.7 19.1 25.9 32.2 30.9 36.9 48.2 46.6 56.2 70.4 66 66.4
